# Spiritual self-care in clinical nursing: An integrative review

**DOI:** 10.1016/j.ijnsa.2025.100407

**Published:** 2025-08-11

**Authors:** Mohammad Salehpour, Sina Sharifi, Akram Parandeh

**Affiliations:** a Student Research Committee, Baqiyatallah University of Medical Sciences, Tehran, Iran; b MSc of Nursing, Department of Geriatric Nursing, School of Nursing and Midwifery, Kermanshah University of Medical Sciences, Kermanshah, Iran; c Nursing Care Research Center, Clinical Sciences Institute, Nursing Faculty, Baqiyatallah University of Medical Sciences, Tehran, Iran

**Keywords:** Self-Care, Spirituality, Nurses, Integrative Review, Spiritual Self-Care

## Abstract

**Background and Aims:**

Spirituality and spiritual self-care are vital for addressing patients’ needs, yet their meaning and scope remain poorly-defined in nursing. We aimed to provide an integrative review of spiritual self-care among clinical nurses.

**Methods:**

This study was an integrative review. Articles were searched using the keywords related to self-care and spirituality in nursing. The search covered the period from 1960 to early March 2024 across databases including PubMed, ISI Web of Science, Scopus, ProQuest, Cochrane Library, and Science Direct, as well as the Google Scholar search engine. The initial search resulted in 12,302 articles. Finally, data were collected and analyzed using the Broome method.

**Results:**

Sixteen studies were included and covered diverse designs, such as qualitative research, reviews, randomized controlled and quasi-experimental studies, opinion articles, and conceptual/theoretical papers. The analysis identified three overarching themes: (1) antecedents, definitions, and dimensions of spiritual self-care; (2) practices; and (3) outcomes, highlighting improvements in nurses’ physical, emotional, social, and spiritual well-being.

**Conclusion:**

We have underscored the critical role of spiritual self-care in enhancing nurses’ well-being and professional development. By clarifying its key antecedents, dimensions, and practices, we have highlighted how spiritual self-care not only strengthened nurses’ physical, emotional, social, and spiritual health but also improved care quality and patient outcomes. These insights may provide a foundation for developing targeted interventions and supportive policies to foster spiritual well-being among clinical nurses.

**What is already known about the topic**

• Spirituality is recognized as an important aspect of holistic nursing care.
• Spiritual self-care is believed to support nurses’ well-being and professional resilience.
• The concept and scope of spiritual self-care in nursing remain inconsistently defined in the literature.

Alt-text: Unlabelled box

**What this paper adds**

• We have clarified the antecedents, dimensions, and practices of spiritual self-care among clinical nurses.
• We have demonstrated that spiritual self-care positively influences nurses’ physical, emotional, social, and spiritual well-being.
• We have highlighted the link between nurses’ spiritual self-care and improved care quality and patient outcomes.

Alt-text: Unlabelled box

## 1 Introduction

Nurses are the largest group of clinical staff in healthcare systems (Aliyari et al., 2019; Ebadi and Khalili, 2014; Salmani et al., 2023). The World Health Organization estimates that around 20 million nurses and midwives are employed worldwide (Williams et al., 2022). They have the most direct contact with patients, providing more than 70 % of care, health, and preventive services. Therefore, the well-being and comfort of nurses can profoundly affect the care delivery process and, consequently, the quality of care provided (Moeini et al., 2011). However, the nursing profession is associated with high psychological stress and significant physical pressures. Nurses work in complex and constantly changing environments and often face a lack of personal autonomy and external support (Janzarik et al., 2022). The National Institute for Occupational Safety and Health in the United States (US) ranks nursing as one of the top 40 most stressful professions globally (Mu et al., 2013). Furthermore, nurses must adopt self-care practices to help them reduce stress (Chipu and Downing, 2022).

The concept of self-care has long been promoted by researchers as an essential need for nurses, not merely to help others but because every nurse deserves to care for themselves (Hawthorne and Barry, 2021). Orem, one of the key theorists on self-care, introduced the concept as part of her theory, asserting that because humans can take responsibility for their health, every individual has the capacity for self-care and can translate this into purposeful self-care roles and behaviors (Rajai et al., 2023). Accordingly, self-care is a learnable behavior that can address many of the needs of patients during illness or health deviations (Peterson and Bredow, 2009). Moreover, the World Health Organization defines self-care as activities that individuals, families, and communities undertake intending to promote health, prevent disease, limit illness, and restore health (World Health Organization, 2024). Self-care is widely recognized as a set of purposeful practices that support physical, mental, social, and spiritual well-being across diverse cultural and professional contexts (Lee and Miller, 2013; Taghlili et al., 2023). Thus, it can be regarded as a holistic approach to self-management, encompassing various dimensions of health.

The World Health Organization recognizes spirituality as one of the dimensions of health and affirms its importance in this regard (World Health Organization, 2013). Spirituality is perceived as the discovery of existential meaning both within and outside oneself (Hawthorne and Barry, 2021). In other words, spirituality is a transcendent or metaphysical phenomenon that is associated with purpose and meaning in life (Green, 2021). In this context, numerous studies have demonstrated that spirituality plays a vital role in individuals’ lives, and addressing the spiritual dimension of care can result in significant differences in physical, psychological, and social outcomes (Dindar et al., 2016; Iri et al., 2019; Taheri et al., 2014).

The term "spiritual self-care" in nursing was first introduced by Jacobson and Burkhardt (1989) and later expanded by White et al. (2010). White et al. (2011) suggested that components of spiritual self-care, such as the ability to make decisions about self-care and the ability to continuously care for oneself, can be enhanced through spirituality, thereby promoting spiritual self-care. Spiritual self-care in nursing involves practices based on spirituality; i.e., spiritual self-care can be considered a set of spirituality-based exercises aimed at enhancing well-being and health. Flickinger (2019) defined nurses’ spiritual self-care as positive physical, mental, and spiritual practices that help increase nurses’ resilience, health, and well-being in response to stressors in the system or life. Moreover, previous researchers have shown that nurses, particularly during the COVID-19 pandemic, used practices such as praying, close contact with nature, meditative walking, relaxation, yoga, and self-awareness to manage self-care (Dezorzi and Crossetti, 2008; Nilsson, 2022). Spiritual self-care can provide nurses with a sense of calm and adaptability, reduce workplace stress and anxiety, and improve quality of care and quality of life (Taghlili et al., 2023).

Antecedents, dimensions, and practices of spiritual self-care are essential components for understanding how spiritual self-care develops and functions in the clinical nursing context. Antecedents help identify the factors and conditions that give rise to spiritual self-care among nurses, offering insight into the contexts that support or hinder its development (Taghlili et al., 2023). Dimensions clarify the core components that define spiritual self-care, helping distinguish it from general self-care or other well-being practices (Ausar et al., 2021). Furthermore, understanding specific spiritual self-care practices used by nurses is crucial for translating this concept into real-world applications (Nilsson, 2022).

Therefore, a starting point for reducing the negative effects of occupational stress and workload among nurses could be adopting health-related spiritual behaviors, including spiritual self-care. Given the importance of spirituality in the health of nurses, who work in a sensitive and highly stressful profession, and considering that studies on spiritual self-care among nurses have received limited attention, our knowledge regarding spiritual self-care practices in nurses is restricted. Furthermore, spirituality and nurses’ focus on spiritual self-care are essential for meeting the spiritual needs of patients (Ausar et al., 2021). We aimed to provide an integrative review of the antecedents, definitions, dimensions, practices, and outcomes of spiritual self-care for clinical nurses. We were guided by the following research questions: (1) What are the antecedents, definitions, and dimensions of spiritual self-care in clinical nursing? (2) What are the key practices of spiritual self-care among nurses? (3) What outcomes are associated with spiritual self-care in clinical settings?

## 2 Materials and methods

We employed an integrative review approach based on the Broome method. Broome's method consists of three stages: literature search, data evaluation, and data analysis. An integrative review was chosen because it allows the inclusion of both empirical and theoretical studies, which is particularly valuable for this topic due to the limited number of empirical studies specifically focused on spiritual self-care in clinical nursing. This approach enables a more comprehensive understanding of the phenomenon by drawing from diverse types of evidence (Whittemore and Knafl, 2005). In the literature search phase, studies are retrieved and then reviewed in four stages according to the inclusion criteria. After meeting the inclusion criteria, the studies are evaluated, followed by data analysis through data reduction, data display, data comparison, and conclusion drawing and verification (Broome, 2000; Whittemore and Knafl, 2005). Inclusion criteria included: relevance to the concept of spiritual self-care among clinical nurses—specifically studies that addressed the antecedents, definitions, dimensions, practices, or outcomes of spiritual self-care, Persian or English language, access to the full text, and meeting at least three out of five criteria from the PRISMA quality assessment checklist used in this review. In addition to original empirical studies, relevant review articles were included. This decision was based on the limited number of empirical studies available on spiritual self-care in clinical nursing. Including review articles allowed the integration of broader theoretical insights and synthesized findings from multiple sources, which enriched the overall understanding of the phenomenon. Exclusion criteria included: (1) studies focused on general self-care without a spiritual component; (2) articles addressing spirituality in patients rather than nurses; (3) papers unrelated to clinical nursing settings; and (4) conference abstracts, editorials, commentaries, or duplicate publications.

Articles were retrieved and extracted from databases including PubMed, ISI Web of Science, ProQuest, Cochrane Library, Science Direct, Scopus, and the Google Scholar search engine, with no time limitations, covering the period from 1960 to early March 2024. The search was conducted using the following keywords and search strategy:

(((((Nurse [Title/Abstract]) OR (Nurses [Title/Abstract])) OR (Nursing [Title/Abstract])) OR ("Registered Nurses" [Title/Abstract])) OR ("Registered Nurse" [Title/Abstract])) AND (((((((((((((("Self-care" [Title/Abstract]) OR ("Self-Management" [Title/Abstract])) OR ("Self-sustenance" [Title/Abstract])) OR ("Self-health" [Title/Abstract])) OR ("Caring for one-self" [Title/Abstract])) OR (Spirituality [Title/Abstract])) OR ("Spiritual self-care" [Title/Abstract])) OR ("Spiritual self-perception" [Title/Abstract])) OR ("spirituality in self-care" [Title/Abstract])) OR ("Spiritual self-care program" [Title/Abstract])) OR ("Nurse spiritual self-care" [Title/Abstract])) OR ("Spiritual self-care of nurses" [Title/Abstract])) OR ("Spiritual self-care of a nurse" [Title/Abstract])) OR ("Spiritual self-care of the nurses" [Title/Abstract])) Filters: Clinical Trial, Meta-Analysis, Randomized Controlled Trial, Review, Systematic Review

**Fig. 1:**
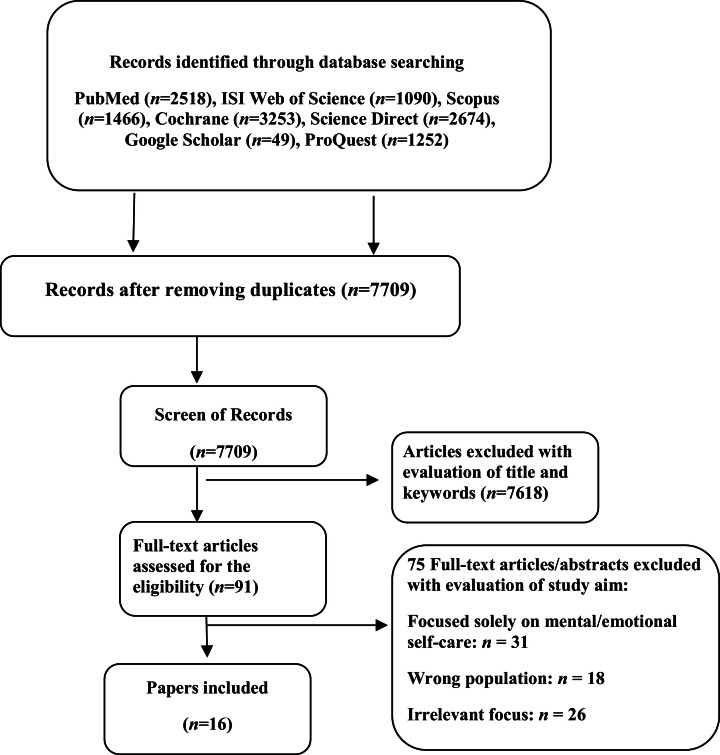
Flowchart indicating the selection of articles through the PRISMA method.

The initial search resulted in 12,302 articles. Several articles were excluded due to being in languages other than Persian or English, conference abstracts, short reports, and studies without author names or publication dates. After carefully reviewing the title, abstract, and full text of many articles, numerous studies were excluded due to differing objectives or irrelevant content, and in the end, 16 articles were included for review (Fig. 1). In the next stage, to evaluate the quality of the articles, five main questions based on the PRISMA quality assessment criteria were considered:

1. Was the study question or objective relevant to the research topic?
2. Was the study method valid?
3. Were the inclusion and selection criteria for articles acceptable?
4. Was the coherence of the collected content acceptable?
5. Were the findings and results reported in a scientifically acceptable manner?

Articles that answered positively to at least three of these five questions were included. In the data analysis stage, the articles were reviewed by two independent researchers throughout the research process. If there was any disagreement between the researchers, a third person was to be consulted, but this was unnecessary as full agreement was reached between the two researchers.

### 2.1 Data extraction process

Data extraction was conducted independently by two authors to enhance reliability and reduce bias. A pre-existing checklist was used to guide the identification and extraction of data related to the antecedents, definitions, dimensions, practices, and outcomes of spiritual self-care for clinical nurses. Each included article was read multiple times by both reviewers to ensure a comprehensive understanding. During the extraction process, no interpretation or paraphrasing was applied; original phrases used by the authors were preserved. The extracted data were then organized and categorized according to the predefined thematic framework.

### 2.2 Statement of ethics approval

This study was an integrative review of previously published literature and did not involve the recruitment of human participants, the collection of primary data, or the use of identifiable personal information. Therefore, ethical approval was not required. All data analyzed in this review were obtained from publicly available sources.

## 3 Results

A total of 12,302 studies were collected from the beginning of 1960 to early March 2024 and reviewed for inclusion criteria based on the three-stage Broome method. In the first stage, 4,593 articles were excluded due to duplication. In the second stage, 7,515 articles were excluded for other reasons, such as unrelated titles and keywords, studies without author names or scientific content, conference abstracts, short reports, lack of full text, or in languages other than Persian or English. In the third stage, 75 articles were excluded after reviewing their abstracts and full texts due to irrelevant content or weak alignment with the study objective. Finally, 16 articles, including three articles in Persian and 13 in English, were included in the study (Fig. 1).

Of the selected articles, five were qualitative studies, three were theoretical or opinion articles, three were review studies, three were experimental studies, and two were quasi-experimental studies. Theoretical and opinion articles were included to strengthen conceptual understanding and to compensate for the limited number of empirical studies available on spiritual self-care in clinical nursing. Additionally, 70 % of the selected studies were published in the past 5 years. Half of the studies (50 %) were conducted in the US, followed by Iran (Table 1). Based on the research question, the findings from the literature review were categorized into three main themes: 1) Antecedents, Definitions, and Dimensions; 2) Practices; and 3) Outcomes of Spiritual Self-Care for clinical nurses.

**Table 1:** Characteristics and main results of studies from the integrated review.

N.	References	Design	Objective	Samples / Regions	Main findings	Limitations
1	Dezorzi and Crossetti, 2008	Qualitative descriptive study	How does spirituality permeate the process of caring for oneself and others in the ICU?	Nine nursing caregivers from the ICU of a university hospital in Brazil.	Spirituality in self-care includes: 1. Prayers; 2. Close contact with nature; 3. The sense of connection with a Higher Power; 4. Self-knowledge.	Small sample size (Nine ICU nurses), limited generalizability beyond the ICU and the Brazilian context.
2	Andrade et al., 2022	Qualitative descriptive study	To understand the actions developed by the Nursing team for the development of spiritual self-care.	Twenty-two nursing professionals from the oncologic hospital in Brazil.	Five categories include: 1. the help of others and the act of caring; 2. social interaction and dialogue; 3. The exercise of religious practices; 4. by the composition of songs; 5. The denial of practices related to spiritual development.	Limited to one oncologic hospital in Brazil; findings may not represent other settings or cultures.
3	Hawthorne and Barry, 2021	Qualitative descriptive study	This article compares spirituality to religion and exemplifies 5 nurses’ spiritual practices during the pandemic.	Five nurses from California, US.	Spiritual practices are examples of resources that can be used effectively in times of stress, including: Touch, Movement, Nourishment, Recognizing and Honoring self.	Very small sample (five nurses) limits generalizability; specific to California nurses during the pandemic.
4	Koren and Papamiditriou, 2013	Qualitative descriptive study	a) to identify the spiritual needs of nurses, b) to understand how nurses experience and use spirituality in their daily work practice, c) to identify facilitators and barriers to supporting the spirituality of the practicing nurse.	11 nurses from a hospital in the US.	Two themes emerged from the data: 1. Care of the patient includes a) meaning and purpose in their work, b) relationships with patients, and c) caring. 2. Care of self includes a) Internal resources (e.g., wisdom gained through the challenges and joys of life), and b) External resources (e.g., mobile phones provided to nurses during working hours that enhanced communication and care delivery).	Small sample (11 nurses) from one US hospital; findings may not apply to other regions or specialties.
5	Rajabipoor Meybodi and Mohammadi, 2021	Qualitative descriptive study	To identify the components of spirituality that affect the resilience of nurses.	11 nurses working in the Corona era from a hospital in Zanjan, Iran.	Seven components derived from the research findings included: Religious values, Ethical orientation, Wisdom, Voluntary activities, Self-awareness, Belief in the other world, Patience, and hope.	Small sample (11 nurses) during the COVID-19 era in one Iranian hospital; results may be context-specific.
6	Nilsson, 2022	Conceptual/Theoretical	To conceptualize and contextualize the terms “spirituality” and “self-care management” as they relate to nursing in particular and holistic health.	Swedish nurses	Three practices are highlighted in this regard: 1. Prayers; 2. Meditative walking; 3. The mindfulness practice of body scanning.	Study based on Swedish nurses only; theoretical/conceptual focus limits empirical application.
7	White et al., 2011	Opinion article	To propose integrating the concepts of spirituality and spiritual self-care within SCDNT.	American nurses	Spiritual self-care is the set of spiritually-based practices in which people engage to promote continued personal development and well-being in health and illness.	Opinion article; lacks empirical data; potential author bias in conceptual framing.
8	Taghlili et al., 2023	A review of concept analysis	To analyze the concept of nurses’ spiritual self-care using Walker's and Avant's approach.	11 studies	Spiritual self-care in nurses is a self-centered care process that leads to more nurses' attention to strengthening the inner spiritual dimensions. It also helps to provide effective care for patients in difficult and stressful professional situations	Review limited by heterogeneity and scope of included studies; possible publication bias.
9	Koren and Purohit, 2014	Integrative Review	To systematically review research that tests spiritual interventions for health care providers.	15 studies	Mindfulness (reiki, meditation, various forms of yoga, relaxation, mantra, and heart touch) was the intervention most widely used.	An integrative review may include studies of variable quality, limited by available literature up to 2014.
10	Ausar et al., 2021	Scoping Review	To comprehensively understand the conceptual definitions, theory, and measurement of nurse spiritual self-care.	10 studies	Definitions: one’s relationship with self, a transcendent relationship beyond the self, and ways of caring for the self.Theories: self-care deficit nursing, holistic nursing, theory of spiritual care, Watson's theory of human caring.Measurements: a) Quantitative measures: The Daily Spiritual Experience Scale, the Spiritual Well-Being Scale, the Nurses’ Spirituality and Delivery of Spiritual Care, the Self-Care Assessment Worksheet, the Spiritual Perspective Scale; b) Qualitative measures: Focus group, philosophical hermeneutic, and structured phenomenological approach; c) c) Mixed Method: self-reported survey and open-ended questions to measure self-care and spiritual self.	A scoping review may not capture all relevant studies, possible selection bias.
11	Abdollahzadeh and Shadin, 2020	Quasi-experimental pre- and post-study	To evaluate the efficacy of spiritual self-care training on nurses' self-compassion and moral courage.	Thirty nurses are working in Pasteur Hospital, Tehran, Iran.	The level of self-compassion and moral courage of the experimental group, compared with the control group, significantly increased.	Quasi-experimental design limits causality inference; sample from one hospital in Iran.
12	Mirzaee et al., 2022	RCT with pre-test and post-test design with a control group	To investigate the effect of spiritual self-care education on the resilience of nurses working in ICUs.	Sixty-four nurses are working in ICUs in Ziaeian Hospital in Tehran, Iran.	Resilience significantly improved in the intervention group following spiritual self-care education compared to the control group.	RCT in ICU nurses, but limited to one hospital; short follow-up period.
13	Bay et al., 2010	RCT with pre-test and post-test design with a control group	To determine whether two 1-day spiritual retreats focused on spiritual well-being as a form of self-care changed a nurse’s spirituality in a positive direction.	One hundred ten nurses from adult ICUs (55 intervention and 55 control) and 121 nurses from a children’s referral hospital (61 intervention and 60 control) in an academic urban critical care hospital in the US.	There was a significant increase in Spiritual Well-Being Scale scores, and an increase in The Daily Spiritual Experience Scale scores between groups over time.	RCT with a specific population (ICU nurses) and a single location; possible Hawthorne effect.
14	Yong et al., 2011	RCT with pre-test and post-test design with a control group	To examine the effect of a spirituality training program on the spiritual well-being, spiritual integrity, leadership practice, job satisfaction, and burnout of hospital middle manager nurses.	Fifty-one nurses (24 nurses in the spirituality program and 27 in the control group) were in the university hospital in Seoul, South Korea.	After the spirituality training program, spiritual well-being, spiritual integrity, and leadership practice improved, and burnout was reduced significantly. The spirituality training program was effective in improving the psychosocial and spiritual well-being of middle manager nurses.	RCT with middle managers only; small sample size; cultural context limits generalizability.
15	Drew et al., 2016	Quasi-experimental pre- and post-study	To evaluate student perceived stress and mindfulness to 1 hour per week of class time is dedicated to mind-body self-care.	One hundred fourteen nursing students from three universities in the US Midwest (50 in the intervention group and 64 in the control group).	There was a statistically significant interaction between intervention and time on Perceived Stress Scale scores. PSS scores of the intervention group decreased from baseline to T3 when the intervention ended, whereas PSS scores of the comparison group increased from baseline. The average scores on the Mindful Attention Awareness Scale did not differ significantly.	Quasi-experimental with nursing students; self-reported measures; limited geographic diversity.
16	Green, 2021	Opinion article	-	Nurses	Spiritual self-care interventions: 1. Reading Scripture; 2. Mindfulness; 3. Prayer; 4. and Self-care time.	Opinion article without empirical evidence; may lack external validity.

ICU: Intensive Care Unit; RCT: Randomized controlled trial; SCDNT: Self-Care Deficit Nursing Theory; US: United States

### 3.1 First theme: antecedents - definitions - dimensions

We found that there were various antecedents for the formation of the concept of spiritual self-care. For example, White et al. (2011) identified the spiritual orientation of nurses as the most essential antecedent for spiritual self-care. Additionally, other studies (Dezorzi and Crossetti, 2008; Green, 2021; Taghlili et al., 2023) highlighted that facing stress and tension is one of the key antecedents of a nurse's spiritual self-care (Table 2). Out of the 16 studies, five focused on definitions, three on dimensions, and one study addressed both aspects together. Definitions of spiritual self-care for nurses were associated with various themes, including **Theology** (referring to belief in God or a higher power), **Self-knowledge** (referring to connection with oneself and alignment with inner strength), and **Other-knowledge** (referring to connection with nature and interaction with others). The theme of theology was explored in studies by White et al. (2011) and Dezorzi and Crossetti (2008), where it was defined as a connection to a higher power, and in the study by Ausar et al. (2021), where it was framed as transcendental (metaphysical) factors. Similarly, the theme of self-knowledge was considered a core aspect of defining spiritual self-care for nurses by Dezorzi and Crossetti (2008). Furthermore, Ausar et al. (2021) and Taghlili et al. (2023) also defined spiritual self-care as achieving inner self-awareness and connecting with oneself. The theme of other-knowledge was also part of the definitions of spiritual self-care, identified as close contact with nature in Dezorzi and Crossetti (2008) and connection with others in White et al. (2011). In this regard, the results of the literature review revealed that the dimensions of spiritual self-care for nurses were multifaceted, encompassing religious (Rajabipoor Meybodi and Mohammadi, 2021; White et al., 2011), ethical (Rajabipoor Meybodi and Mohammadi, 2021), psychological-emotional (Koren and Purohit, 2014; Rajabipoor Meybodi and Mohammadi, 2021), social (Koren and Purohit, 2014; Rajabipoor Meybodi and Mohammadi, 2021; White et al., 2011), intrapersonal (self-knowledge), and interpersonal (other-knowledge) dimensions (Table 2).

**Table 2:** Antecedents, definitions, and dimensions of spiritual self-care for clinical nurses.

N.	References	Dimensions	Antecedents	Definitions
**1**	White et al., 2011	1. Connection with a higher power (e.g., personal prayers, reading sacred texts, participating in religious ceremonies, group worship, or other religious traditions). 2. Connection with others (e.g., improving personal relationships, participating in group activities, building social and volunteer networks).	• Family upbringing • Ethical and religious background • Spiritual orientation • Life experiences rooted in faith, emotions, and feelings	Spiritual self-care is a set of spirituality-based practices aimed at supporting personal growth and well-being across physical, emotional, and spiritual domains.
**2**	Dezorzi and Crossetti, 2008	-	• Work-related tension and stress • Facing experiences of patient death and pain • Support from colleagues	Spiritual self-care is defined as a set of daily practices that foster connection with nature, self-awareness, and a relationship with a higher power, such as praying and seeking self-knowledge.
**3**	Taghlili et al., 2023	-	• Facing intense pressure and stress • The Spiritual nature of the nursing profession • Spirituality-centered workplace	Spiritual self-care is the integration of inner self-reflection and outward spiritual expression, including self-awareness, connection with a higher power, and engagement in religious rituals such as prayer and reading sacred texts.
**4**	Green, 2021	-	• Stress caused by the COVID-19 pandemic	Spiritual self-care refers to intentional practices such as mindfulness, prayer, and sacred reading that support the renewal and maintenance of nurses’ physical, mental, and spiritual well-being.
**5**	Ausar et al., 2021	-	-	Spiritual self-care is a multidimensional concept that includes transcendent factors (such as connection with the cosmos, universe, or a higher power), inner-related factors (like mindfulness, resilience, and inner peace), and personal growth elements (such as self-assessment and protective coping strategies).
**6**	Rajabipoor Meybodi and Mohammadi, 2021	Religious values (having a higher purpose, altruism with patients, and serving others). Ethical values (humility, honesty with oneself and others). Wisdom (gaining knowledge and commitment to ethical and religious principles). Volunteer activities (striving for a higher purpose and engaging in charitable acts). Self-awareness (recognizing strengths, weaknesses, emotions, and resilience levels). Belief in the hereafter (faith, practical religiosity, and rejection of materialism). Patience and hope (maintaining a positive outlook, perseverance in challenges, and empathy with patients).	-	
**7**	Koren and Papamiditriou, 2013	Intrinsic motivation: Reflection and decision-making, such as gaining experience and wisdom through life's ups and downs. External resources: Facilitators: Use of mobile phones, daily discussions and interactions, the blessing of the hand’s ceremony, Nurses Week, memorial services, and debriefing sessions. Inhibitors: Greater organizational presence, the existence of specific online social networks for nurses, daily conversations with colleagues, and support from spiritual guides and individuals	-	-

### 3.2 Second theme: spiritual self-care practices

Ten of the selected studies specifically addressed spiritual self-care practices for nurses. These practices generally fell into two categories: Religious and spirituality-based lifestyle practices, Relaxing body movement and mindfulness-based practices, and Theoretical Frameworks Informing Spiritual Self-Care Practices.

#### 3.2.1 Religious and spirituality-based lifestyle practices

This category included activities such as prayer, reliance on God, forgiveness, patience, and gratitude, intended to foster spiritual well-being and resilience. For example, two studies (Abdollahzadeh and Shadin, 2020; Mirzaee et al., 2022) emphasized incorporating daily spiritual rituals and religious beliefs into personal care routines. Another study (Bay et al., 2010) described spiritual retreats involving meditation, relaxation, nature walks, and listening to music as part of a structured spiritual self-care program.

#### 3.2.2 Relaxing Body Movement and Mindfulness-Based Practices

Several studies focused on mindfulness and meditative practices to help nurses manage stress and improve self-awareness. Two studies (Hawthorne and Barry, 2021; Koren and Purohit, 2014) reported on mindfulness-based practices that promoted non-judgmental awareness of present emotions. A spiritual training program based on *mantram* repetition (a short, powerful, and spiritual phrase) was discussed in one study (Yong et al., 2011), while the Urban Zen Integrative Therapy program, incorporating yoga, meditation, aromatherapy, and Reiki, was presented in another (Drew et al., 2016). These interventions were often designed to calm the mind and body and enhance resilience.

#### 3.2.3 Theoretical frameworks informing spiritual self-care practices

In addition to the above practices, some studies used theoretical models to support the development or integration of spiritual self-care practices. For example, Orem’s Self-Care Deficit Nursing Theory (White, 2010; White and Schim, 2013) provides a conceptual foundation for understanding the role of self-care in nursing and has been adapted to incorporate spiritual components. One study (White et al., 2011) proposed integrating spiritual self-care into Orem’s theory as a critical step toward enhancing holistic nursing care Table 3.

**Table 3:** Spiritual self-care exercises for clinical nurses.

References	Approach	Theoretical Foundations	Exercises
Abdollahzadeh and Shadin, 2020	**Religious and Spirituality-Based Lifestyle Practices**	Richards and Bergin's Spiritual Model	• Reliance on and appeal to God • Prayer and supplication • Gratitude • Patience
Mirzaee et al., 2022			• Concentration and meditation • Muscle relaxation and deep breathing • Prayer and supplication • Reliance on God • Altruism and helping others • Seeking forgiveness and amending past mistakes • Forgiveness of others and self-compassion • Gratitude to God and others • Importance and role of patience
Bay et al., 2010		Spiritual Retreat Program	• Meditation • Muscle relaxation • Nature walks • Yoga • Journaling • • Music
Hawthorne and Barry, 2021	**Relaxing Body Movement and Mindfulness-Based Practices**	Mindfulness-based intervention or Conscious Body Awareness	• Touch-based practices (e.g., hydrotherapy or oil therapy) • Movement practices (e.g., mindful breathing, meditation, mindfulness, yoga, and connecting with nature, sea, and plants) • Healthy nutrition (e.g., vegetarian or low-fat diets, avoiding refined sugars, and reducing pesticide exposure) • Self-recognition and silence
Koren and Purohit, 2014			• Kabat-Zinn mindfulness exercises, including yoga, meditation, and Qigong • Reiki or energy healing, Tai Chi, yoga, and meditation • Progressive muscle relaxation • Heart-touch meditation • Mantra meditation
Yong et al., 2011		Meditation	• Selecting a sacred word or phrase from various religious traditions • Repeating the sacred word or phrase • Relaxation and slowing down • Focus on a specific goal • Integration of these practices
Drew et al., 2016		Urban Zen Integrative Therapy Program (UZIT)	• Yoga • Aromatherapy • Reiki or energy therapy • Mindful breathing • Contemplative caregiving
Nilsson, 2022		-	• Mindful body scanning • Prayer • Meditative walking
Andrade et al., 2022		-	• Caring for others through empathy, attention, love, and caregiving • Social interaction (psychosocial dimension) and conversations with psychologists, family members, and social groups through daily socialization • Religious practices (e.g., reading sacred texts, praying, listening to music, watching films, and attending religious gatherings) • Songwriting and hymn singing
White et al., 2011	**Theoretical Frameworks Informing Spiritual Self-care Practices**	Orem's Self-Care Deficit Nursing Theory	• Listening to music • Meditation • Yoga or Tai Chi • Reading inspirational works • Appreciating nature through walks, hiking, or quietly sitting by riversides or meadows

### 3.3 Third theme: outcomes of spiritual self-care

According to Table 4, the outcomes of spiritual self-care for nurses were divided into two dimensions: **Personal** and **Professional**. Spiritual self-care enhanced nurses' overall health across four key dimensions derived from the included studies: physical health (e.g., reduction in pain and physical tension) (Koren and Purohit, 2014; Nilsson, 2022), psychological-emotional health (e.g., resilience, well-being, and hope) (Abdollahzadeh and Shadin, 2020; Dezorzi and Crossetti, 2008; Rajabipoor Meybodi and Mohammadi, 2021), social health (e.g., improved interactions with patients and increased fellowship among colleagues) (Drew et al., 2016; Hawthorne and Barry, 2021; Koren and Papamiditriou, 2013; Koren and Purohit, 2014), and spiritual health (e.g., spiritual coping, spiritual awareness, and spiritual integrity) (Ausar et al., 2021; Taghlili et al., 2023; Yong et al., 2011). These findings are consistent with the World Health Organization’s holistic view of health and reflect the multidimensional impact of spiritual self-care on nurses’ well-being. These effects contributed to achieving existential well-being and peace (Nilsson, 2022; Taghlili et al., 2023; White et al., 2011). Furthermore, adopting spiritual self-care behaviors for nurses was identified as a turning point in delivering high-quality and effective care to patients (Ausar et al., 2021; Taghlili et al., 2023).

**Table 4:** Spiritual self-care outcomes for clinical nurses.

Dimensions	Outcomes
**Personal**	• Reduction in pain and physical tension • Inner peace • Emotional well-being • Resilience • Hope • Understanding meaning and purpose in life • Discovering inner (self) strength • Flexibility • Control of negative thoughts and emotions • Spiritual adaptation • Spiritual awareness • Spiritual well-being • Spiritual integrity • Enhanced sense of well-being • Increased self-awareness
**Professional**	• Increased motivation at work • Professional growth • Increased empathy and solidarity among colleagues • Reduced burnout • Strengthened professional commitment • Open communication with nursing managers • Improved care quality • Enhanced interactions with patients and colleagues • Managing stress in the workplace • Protection against fatigue and work pressure

## 4 Discussion

We aimed to conduct an integrative review of spiritual self-care for clinical nurses.

One part of the findings from this study was related to the examination of the antecedents, definitions, and dimensions of spiritual self-care for nurses. Among the antecedents, the spiritual orientation of nurses emerged as the most essential and recurring factor across the reviewed literature. While professional stress and its recognition in the workplace were also frequently mentioned, these appeared more as contributing conditions that prompted the need for spiritual self-care rather than its foundational antecedent. In this regard, Taghlili et al. (2023), in their review, concluded that enduring work pressure and difficult experiences in the workplace allowed nurses to express their inner selves and, ultimately, enhance their spiritual selves. Similarly, Green (2021), in a study conducted during the COVID-19 pandemic, found that the spread of COVID-19, as an unknown stressor, could lead nurses to renew and maintain their spiritual health (Green, 2021). Generally, Hawthorne and Barry (2021) asserted that when individuals face a stressful life event, maintaining their own and their family's health becomes a life goal; thus, nurses may resort to strategies, including strengthening their spirituality, as a way to cope. Researchers have found that if spirituality is used as a strategy, it can help individuals understand their experiences of suffering and pain, instill hope and peace, promote a sense of inner coherence, and bring spiritual healing (Dezorzi and Crossetti, 2008; Nilsson, 2022). Therefore, spirituality has been identified as a foundational element in achieving a comprehensive understanding of spiritual self-care for nurses. In this study, spiritual self-care was conceptualized through three interrelated themes: self-knowledge, other-knowledge, and theology (God-knowledge). These dimensions are not necessarily experienced in a fixed sequence but may emerge simultaneously or dynamically, depending on individual experiences and contexts. For example, nurses may begin by cultivating inner awareness and personal reflection (self-knowledge), which can support meaningful interactions with others and the environment (other-knowledge), and foster a sense of connection with a higher power (theology). Dezorzi and Crossetti (2008) also emphasized self-knowledge as a core aspect of spirituality and spiritual self-care for nurses and highlighted the role of connection with nature, the universe, and a transcendent power in achieving spiritual well-being. Moreover, Ausar et al. (2021) and Taghlili et al. (2023), through review studies, noted that attention to self (internal factors) and beyond self (transcendental and cosmic factors) were key concepts of spiritual self-care for nurses. Thus, based on the available antecedents and definitions, it can be concluded that spiritual self-care for nurses is multifaceted and can include dimensions such as religious, ethical, psychological-emotional, and social aspects. However, no study was found that exclusively focused on the dimensions of spiritual self-care for nurses. Nonetheless, the results of other similar studies were reviewed and discussed. For example, Rajabipoor Meybodi and Mohammadi (2021) in their qualitative study identified seven spiritual components affecting the resilience of Iranian nurses, including religious values, ethical values, self-awareness, patience and hope, wisdom, voluntary activities, and otherworld beliefs. Similarly, White et al. (2011) identified two general dimensions: connection with a higher power (religious dimension) and connection with others (social dimension). The multifaceted nature of spiritual self-care for nurses, along with its emphasis on high human values, could lead to increased social participation, the development of resilient behaviors, and enhanced adaptability. Nurses, as they face various psychological tensions and emotional distress, also have unique spiritual needs that, when addressed through different methods, can lead to an improvement in their quality of life (Taghlili et al., 2023).

In the second section, we focused on spiritual self-care practices for nurses. There were two main approaches: religious and spirituality-based lifestyle practices and relaxing body movement and mindfulness-based practices, supported by various theoretical frameworks. The religious lifestyle approach emphasized the spiritual and religious aspects of health, encouraging balance between body, mind, and spirit for overall well-being. For example, Abdollahzadeh and Shadin (2020) demonstrated that spiritual self-care enhanced self-control, stress management, and attention to religious values, leading to increased self-compassion among nurses. Similarly, Mirzaee et al. (2022) reported significant improvements in resilience following a spiritual self-care educational intervention. These findings may reflect many societies where religious beliefs are intertwined with a culture that would affect lifestyle; for example, the cultural context of Iranian society, whereby religious beliefs strongly influence lifestyle and health behaviors. (Mashalchi Zade et al., 2024). On the other hand, the majority of researchers focused on relaxing body movement approaches to spiritual self-care, which share a common goal of promoting awareness, inner peace, and stress reduction. These approaches are grounded in meditation-related practices that integrate mind and body to improve mental focus and emotional regulation. The term "meditation" focuses on the integration of mind and body and is used to calm the mind and improve the quality of life. Meditation refers to a series of calming mental exercises that individuals perform to relieve anxiety and tension. This method uses mental and physical techniques to focus on mindful breathing and help individuals achieve relaxation in various ways (Alhawatmeh et al., 2022). For example, Yong et al. (2011) developed interventions based on meditation concepts specifically for nursing managers and found that the implementation of a spirituality-based educational program centered on meditation practices enhanced spiritual integration, improved leadership skills, and reduced burnout, thereby promoting the psychological, social, and spiritual well-being of nursing managers. Similarly, Koren and Purohit (2014) found that most of the studies they examined were based on mindfulness. They indicated that the themes emerging from these studies were all positive, showing that spirituality-based interventions led to increased self-awareness, enhanced mental focus, improved physical performance, reduced stress and anxiety, and better relationships with patients and colleagues. Thus, it can be concluded that the results of the present study align with evidence suggesting a direct and effective connection between spiritual self-care practices and the well-being and peace of individuals. Other practices, including spiritual retreats, gentle body movements like yoga, and integrative therapies such as Urban Zen Integrative Therapy, further contributed to relaxation and well-being (Von Visger et al., 2024). These body-mind interventions provided accessible and practical methods for nurses to engage in spiritual self-care, helping them cope with professional stress and maintain holistic health. Overall, these findings suggest that while religious lifestyle practices remain culturally significant, relaxing body movement approaches represent a broader, versatile set of spiritual self-care strategies that positively impact nurses' health and well-being.

In the final section, we focused on the outcomes of spiritual self-care for nurses. These outcomes are events that lead to the emergence of a concept (Walker and Avant, 2005). Accordingly, the consequences of spiritual self-care for nurses were divided into two dimensions: personal and professional. In the present study, the personal outcomes of spiritual self-care were positive results that contributed to the improvement of the physical, psychological, and spiritual well-being of nurses. The professional outcomes, on the other hand, involved the enhancement of their social health. Physical health refers to the ability to maintain a healthy quality of life, allowing individuals to engage in daily activities without excessive fatigue or physical stress (Hjelm, 2010). In this regard, Nilsson (2022) also highlighted better control of physical stress as one of the consequences of spiritual self-care for nurses. Moreover, Koren and Purohit (2014) considered positive physical changes, such as reduced physical pain, as benefits of spirituality-based interventions. On the other hand, psychological well-being is an inner positive outlook where an individual is psychologically equipped to cope with daily stressors and, ultimately, can provide effective care (Hjelm, 2010). From the results, we have indicated that spiritual self-care for nurses can enhance resilience and promote a sense of inner peace. Ausar et al. (2021), Dezorzi and Crossetti (2008), Mirzaee et al. (2022), Rajabipoor Meybodi and Mohammadi (2021), Taghlili et al. (2023), and White et al. (2011), in both international and national studies, all agreed on this and confirmed these findings. Similarly, spiritual well-being is defined as living a purposeful and meaningful life, engaging effectively with others, and believing in a higher power (Mashalchi Zade et al., 2024). In line with this, researchers conducting a literature review showed that the promotion of health and strengthening spiritual well-being are key outcomes of spiritual self-care for nurses. Bay et al. (2010) and Yong et al. (2011) in their studies stated that spirituality-based practices for nurses' self-care led to awareness and spiritual well-being. Spirituality, being intrinsic to spiritual self-care and serving as a necessary precursor to its formation, can lead to both spiritual well-being and health for nurses. Additionally, the other side of the consequences of spiritual self-care for nurses involves enhancing their social health and well-being, which were categorized under professional outcomes. Social health refers to the creation and maintenance of healthy relationships and meaningful, proper interactions with others (Hjelm, 2010). For example, in the present study, increased motivation at work, enhanced empathy and solidarity among colleagues, improved interactions with patients, and stress management in the workplace were all mentioned as examples of social well-being. By engaging in spiritual self-care practices and applying them in stressful situations, nurses may achieve inner growth and integration. This may create a strong energy field between the nurse and the patient, ultimately leading to improved quality of life and social well-being (Ausar et al., 2021; Murgia et al., 2020).

### 4.1 Limitations

This study has several limitations. First, there was limited access to the full text of some relevant articles, which may have restricted the comprehensiveness of the data. Second, the search was limited to studies published in English and Persian, potentially excluding valuable findings from studies published in other languages. Third, only one of the included studies explicitly employed a theoretical framework, limiting the ability to systematically compare theoretical underpinnings across studies. Fourth, the integrative review design itself, while broad and inclusive, may introduce bias due to the diversity of methodologies among the included studies. Additionally, potential publication bias cannot be ruled out, as studies with significant or positive results are more likely to be published and accessible. Finally, the majority of the included studies were qualitative and conducted in diverse cultural and clinical contexts, including Brazil, the US, Iran, South Korea, and Sweden. While this diversity enriches the understanding of spiritual self-care, it also introduces heterogeneity that limits direct comparisons and generalizability of findings.

## 5 Conclusion

It can be concluded that the main antecedent for nurses’ spiritual self-care was their spiritual orientation, which can vary in different societies. Moreover, spiritual self-care has a multidimensional concept and not only contributes to the physical, psychological, social, and spiritual health of nurses but also enhances their well-being, peace, and existential calm, becoming a turning point in providing high-quality and effective care to patients. Therefore, the results of this study could help emphasize the inclusion of spiritual self-care topics in the nursing student curriculum, as well as encourage policymakers and clinical education planners to consider these concepts and their effects when developing educational content for nurses. Given the limited number of studies and theoretical frameworks available on this topic, further research is needed to deepen understanding of spiritual self-care in nursing practice and to support the development of culturally sensitive interventions and educational programs.

## Statement of ethics approval

This study was an integrative review of previously published literature and did not involve the recruitment of human participants, the collection of primary data, or the use of identifiable personal information. Therefore, ethical approval was not required. All data analyzed in this review were obtained from publicly-available sources.

## CRediT authorship contribution statement

**Mohammad Salehpour:** Writing – review & editing, Writing – original draft, Visualization, Methodology, Investigation, Conceptualization. **Sina Sharifi:** Writing – review & editing, Writing – original draft, Validation, Methodology, Investigation. **Akram Parandeh:** Writing – review & editing, Writing – original draft, Methodology, Investigation, Conceptualization.

## Declaration of competing interest

The authors declare that they have no known competing financial interests or personal relationships that could have appeared to influence the work reported in this paper.
